# Potential savings through single-dose intravenous Dalbavancin in long-term MRSA infection treatment – a health economic analysis using German DRG data

**DOI:** 10.3205/id000043

**Published:** 2019-10-23

**Authors:** Michael Wilke, Kerstin Worf, Birgit Preisendörfer, Wolfgang Heinlein, Tilman Kast, Klaus-Friedrich Bodmann

**Affiliations:** 1 inspiring-health GmbH, Munich, Germany; 2 Klinikum Barnim GmbH, Werner Forßmann Krankenhaus, Eberswalde, Germany

**Keywords:** single-dose intravenous Dalbavancin, health economic model, length of stay, cost savings

## Abstract

Complicated infections such as osteomyelitis, skin and soft tissue infections or endocarditis often require antibiotic therapies that can last up to several weeks. The prolonged hospital length of stay (LOS) leads to a dramatic increase in costs. Single-dose intravenous Dalbavancin is a novel antimicrobial agent for the treatment of acute bacterial skin, skin structure and soft tissue infections (ABSSSI) that allows an earlier discharge of patients, resulting in potential savings. Joint, bone and prostheses infections (JBPI) are also related with long LOS. The aim of this study is to determine the economic effects of single-dose intravenous Dalbavancin in suitable patients with Methicillin-resistant *Staphylococcus aureus* infections in Germany. For this purpose, an analysis with real-world patient treatment data was performed, which was subsequently validated in a large German hospital. In total, ABSSSI patients with MRSA infections could stay 6.45 days shorter and 2,865 € could be saved while JBPI patients could be discharged eventually 10.6 days earlier and 3,909 € could be saved. Single-dose intravenous Dalbavancin is thus an option for patients with ABSSSI and JBPI who are eligible for discharge.

## Introduction

Complicated infections often require a long antibiotic therapy period. Especially in diseases such as osteomyelitis, skin and soft tissue infections or endocarditis, antibiotic durations up to 3–6 weeks are possible [1], [2], [3], [4], [5], [6], [7], [8]. Patients with Methicillin-resistant *Staphylococcus aureus* (MRSA) infections stay in hospital for even longer [9], [10]. As hospitals in Germany are funded by diagnosis-related group (DRG) payments per case, the length of stay (LOS) has become a critical economic factor [11], [12], [13], [14], [15], [16]. With this in mind, reducing the LOS seems to be an economically viable option. Studies show that early discharge and outpatient continuation of antibiotic therapy, for example by oralization, may lead to savings [17], [18], [19], [20]. Oralization is often accompanied by problems related to patients’ compliance. Other problems are that sometimes because of intolerance or resistance, no oral option is available. With Dalbavancin, however, a novel antimicrobial agent is available that enables an intravenous single-dose with an extremely extended half-life in Gram-positive bacteria. Dalbavancin thus gives clinicians the opportunity to provide an antibiotic that is proven to be as effective as conventional therapies without the need for prolonged hospitalization drastically reducing the LOS and the total cost per patient [21]. The aim of this study is to determine the economic effects that could be achieved by single-dose intravenous Dalbavancin in suitable patients with MRSA infections in Germany.

## Methods

In Germany, inpatient cases are reimbursed using a fee-per-case system (DRG system) based on the principle “same money for same service”. This poses a challenge for hospitals as cost-covering performance can only be provided up to a certain point in time after which the costs exceed the revenues. The calculation basis of the Institute for Hospital Reimbursement (InEK) is the average cost per DRG of all hospitals participating in this calculation. For each DRG, LOS thresholds are defined as the lower trimpoint LOS (ltpLOS), average LOS (aLOS) and upper trimpoint LOS (htpLOS) [16], [22], [23], [24]. If the average LOS is exceeded, the patient's LOS represents a cost driver for the hospital within the DRG system. Patients with MRSA infections often have significantly longer LOS and are therefore above the average lengths of hospital stay. Exceeding the LOS is associated with increased costs, which is why these patients cause a loss for the hospital [25], [26], [27].

In order to evaluate possible cost savings, an economic model was developed based on the assumption that there is a cohort of patients with MRSA infections who are basically dischargeable but only remain in hospital for the administration of a necessary intravenous antibiotic therapy. Using the example of Dalbavancin, an antimicrobial agent for the treatment of acute bacterial skin, skin structure and soft tissue infections (ABSSSI) in gram-positive pathogens, it was calculated to what extent a hospital could benefit from shortening the inpatient stay of suitable patients.

With a terminal half-life of >14 days, Dalbavancin can be administered via an intravenous single-dose of 1,500 mg [28] which enables the hospital to discharge the patient under the protection of an optimal antibiotic therapy. Dalbavancin thus provides a dosing regimen with infrequent parenteral administration to treat infectious diseases that otherwise require daily intravenous therapy for many weeks [28]. To estimate possible savings through single-dose intravenous Dalbavancin in patients with MRSA infections, clinical entities that are often associated with MRSA infections and that bare the option of early discharge were described. For the ABSSSI group, we used the FDA definitions [29]. On top of these, we defined a group of joint, bone and prostheses infections (JBPI), where infection treatment is often long and single-dose administration could be a tool for shortening LOS in hospital. Table 1 (Tab. 1) shows the classification of the entities. A table that includes the ICD-10 codes used is provided in Attachment 1.

As infections and especially infection with MRSA negatively impact the LOS of patients, we needed data to compare the aLOS of the DRG – which is always a mix of patients with no infections, with infections and with MRSA infections as most DRGs are not specifically designed for infection patients – with the LOS of infection patients and MRSA infection patients in the respective infection groups. This analysis was done on a large benchmarking database, in which 300 hospitals participated and about four million cases are registered annually. In order to understand the variation, all cases in the above-mentioned infection groups from 2016 were analysed [30].

To assess possible savings, a 3:1 propensity score matching was performed. Matched were the infection cases with MRSA infection cases against infections without MRSA from the two groups (ABSSSI and JBPI) using age, sex, principle diagnosis, DRG and comorbidities (via Charlson Comorbidity Index – CCI) in order to control confounders for costs, using a caliper of 0.1. The difference in costs and aLOS were then analysed.

According to the literature, the proportion of patients with ABSSSI who could be discharged earlier through oralization is 37.9 percent [17]. Within the model, it was assumed that the same proportion of patients who could potentially receive single-dose intravenous Dalbavancin also applies to wound infections. Therefore we assumed that 37.9 percent of all MRSA patients could benefit from single-dose intravenous Dalbavancin.

The difference in costs between infections without and with MRSA was taken as possible savings. The total result was calculated by taking into account these savings as well as adding the treatment costs (2,280 € per 1,500 mg dose).

After the analysis the proportion of patients and the shortening of LOS were validated by a case-review in a large major urban hospital.

## Results

Case data from 171,074 patients in the respective infection groups have been analysed. Table 2 (Tab. 2) shows the distribution of cases before and after 3:1 propensity score matching. 

The results of detailed analysis per infection group are shown in Table 3 (Tab. 3) and Table 4 (Tab. 4).

The analysis of real-world cases shows that in ABSSSI the presence of MRSA leads to excess costs of 5,145 € and a prolongation in LOS of 6.45 days, while in JBPI the excess costs of MRSA are even higher with 6,189 € and additional LOS of 10.59 days. Overall, the use of single-dose intravenous Dalbavancin and early discharge has the potential to create an average saving of 2,964 € as shown in Table 5 (Tab. 5).

The weighted LOS difference in ABSSSI and JBPI was 6.8 days.

The peer review of MRSA infection cases in a major urban hospital was conducted to validate the results of the data analysis. Of 211 MRSA patients, 108 were carriers and had no infection. Of the remaining 103 cases, 47 patients had underlying conditions that would potentially make them suitable for single-dose intravenous Dalbavancin and subsequent discharge. Of these 47 cases, 32 percent could have been released. In average, LOS would have been 8.5 (varying from 4–18) days shorter. The savings calculated in this patient group were 2,281 €.

## Discussion

The health economic analysis shows that cost savings can be achieved by administering single-dose intravenous Dalbavancin in patients with MRSA infections. The results are in line with other studies that have investigated the economic effects of Dalbavancin [31], [32], [33]. Single-dose intravenous Dalbavancin shortens the LOS and thus results in cost savings. 

MRSA incidence was 10.8 percent for ABSSSI and 11.3 percent for JBPI. The incidence of MRSA has remained steady in German hospitals. A slight decline from 0.71 percent in 2013 to 0.69 percent in 2016 was recorded for the overall MRSA rate in Germany according to Destatis. Nevertheless, 131,014 of 18,959,832 inpatients in Germany had MRSA as a pathogen coded [34]. The Antibiotic Resistance Surveillance (ARS) at the Robert Koch Institute (RKI) determined an MRSA prevalence of 12.1 percent in 2015 and 10.6 percent in 2016 for blood cultures from inpatient care [35]. Although national MRSA percentages vary widely between 1.2 percent and 50.5 percent [36], MRSA isolation in ABSSSI can be as high as 25 percent in Europe [37]. Our findings are in line with other sources.

Although outpatient parenteral antibiotic therapy (OPAT) has not yet been established in Germany, it provides a theoretical option for avoiding or reducing an inpatient stay. However, it cannot be considered appropriate for all patients as some of them may be unable to travel to receive their treatment [38], [39], [40]. Likewise, the possibility of oralization is not suitable for all patients who are eligible for discharge. A key factor here is the patients’ compliance. Studies show that the compliance of patients depends on various factors and is often low [41], [42], [43]. For example, Eells et al. state that patient adherence to oral antibiotic therapy for SSTI after hospital discharge was only 57 percent, which was associated with poor clinical outcomes [43]. Thus, a single-dose intravenous Dalbavancin is an option for patients where the LOS can be significantly reduced.

Routine data was used to calculate savings estimates. These were derived from the cost difference of patients with or without MRSA infection. This is admittedly a crude estimate and may differ in clinical reality. The validation in a major urban hospital showed that 32% of the patients were eligible for discharge. This is less than the findings of Eckmann et al. [17], who reported a discharge opportunity rate of 47 percent in Germany and 37.9 percent in the entire study. However, the peer-review of the hospital cases carried out in the context of this paper revealed possible savings of 8.5 days, while Eckmann et al. found savings of only 6.2 bed days per case. This implies that findings will probably vary from hospital to hospital. 

The achievable savings in the validation group of 2,281 € were close to our calculation of 2,964 €. This can be an indicator that the estimates used were solid. However, for compliant patients that tolerate oral antibiotics the savings would be 5,244 € in average. That again is close to the measured cost differences in the two groups of ABSSSI and JBPI. The savings are calculated using the average cost difference. Therefore no 95% CI for the calculated effect is available. The challenge in calculating savings is to pick the right patients. The assumption to apply the possible average savings to 37.9% of all MRSA patients is currently investigated in a prospective evaluation of the use of Dalbavancin i.v. in a tertiary care hospital.

Moreover, extended LOS in hospital is known to be an independent risk factor for the increase of hospital acquired infections [44], [45], [46]. Therefore, reducing LOS through early discharge has clinical and economical benefits.

Applying antibiotics with a half-life of 14 days or more can imply a risk for possible adverse effects. The available data on Dalbavancin show a good safety profile with nausea (2.4 %), diarrhoea (1.9 %), and headache (1.3 %) being the only common side effects according to SmPC and generally of mild or moderate severity [47]. A meta-analysis by Dunne et al. showed less treatment-related adverse events and severe adverse events than comparators from phase 2/3 clinical trials with agents including vancomycin, linezolid, cefazolin, nafcillin, or oxacillin. The duration of adverse events proved to be not longer than with short-acting antibiotics (median 3.0 days Dalbavancin vs. 4.0 days comparators), nor did it take longer until the adverse events occurred (median 3.0 days Dalbavancin vs. 3.0 days comparators) [48].

## Conclusion

The LOS of patients with MRSA ABSSSI and JBPI infections is substantially higher than in patients with infections without MRSA in the respective DRGs and causes significant extra costs. Therefore, single-dose intravenous Dalbavancin may be an attractive option for dischargeable patients with MRSA infections who would otherwise be ineligible for OPAT or oralization due to factors such as lack of social support, frailty or substance misuse [40], [49], [50], [51], [52], [53]. Patients with an expectedly long intravenous antibiotic therapy (6–10 days) and no other reason to stay in hospital could be selected.

## Notes

### Author contributions

MW developed the general idea of the calculation and defined the entities in scope together with KFB. KW did the initial literature research, the statistical analysis and prepared the draft of the publication. BP undertook all queries at the German Federal Statistical Office. WH has compiled the model and all data for the calculations. TK did the additional literature research for the publication. KFB acted as clinical advisor and carried out the peer review together with MW.

### Competing interests

The authors received an unrestricted research grant from Correvio GmbH. The sponsor had no influence on the design of the model, the DRG and case selection or the text of the publication. 

## Supplementary Material

Definitions for infection groups and infections with corresponding ICD-10 codes

## Figures and Tables

**Table 1:**
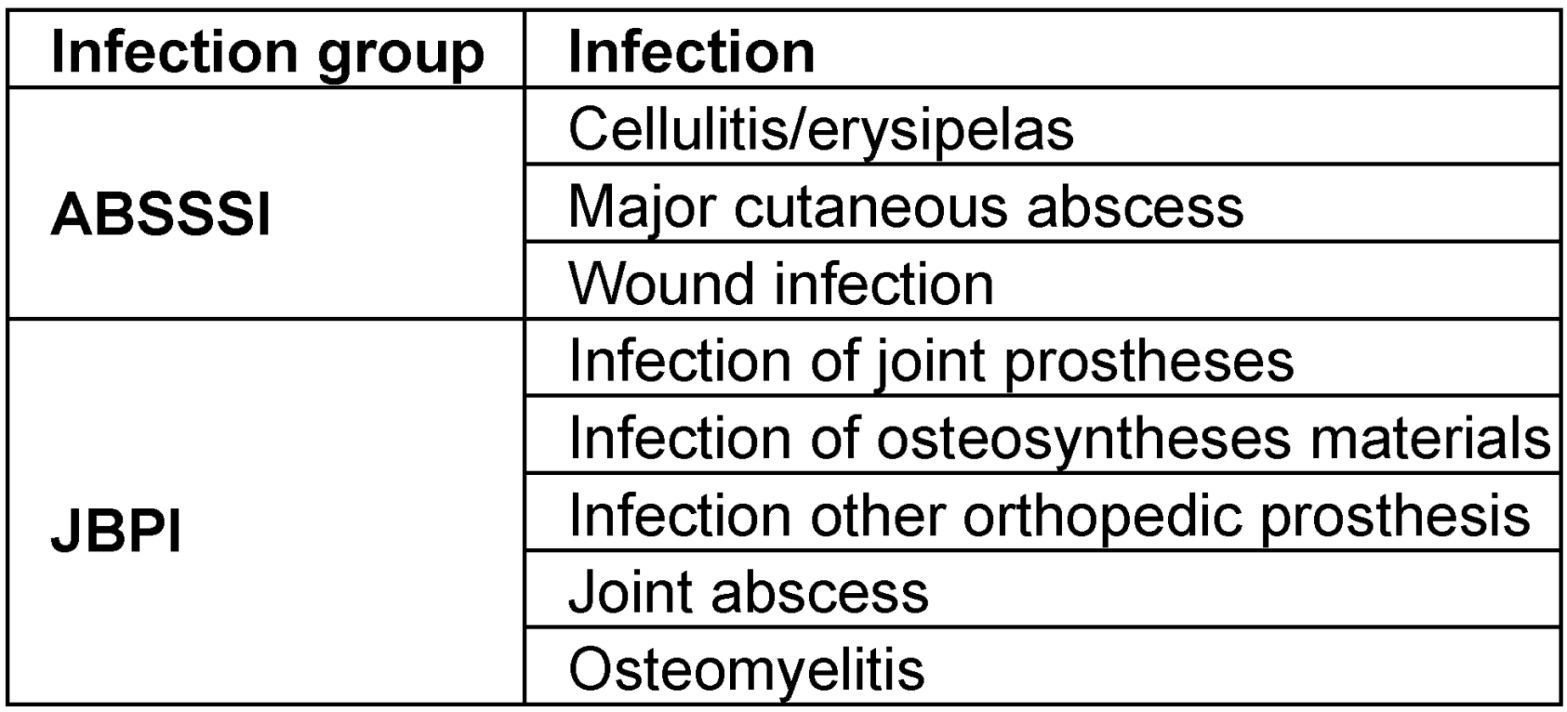
Overview of infection entities for analysis

**Table 2:**
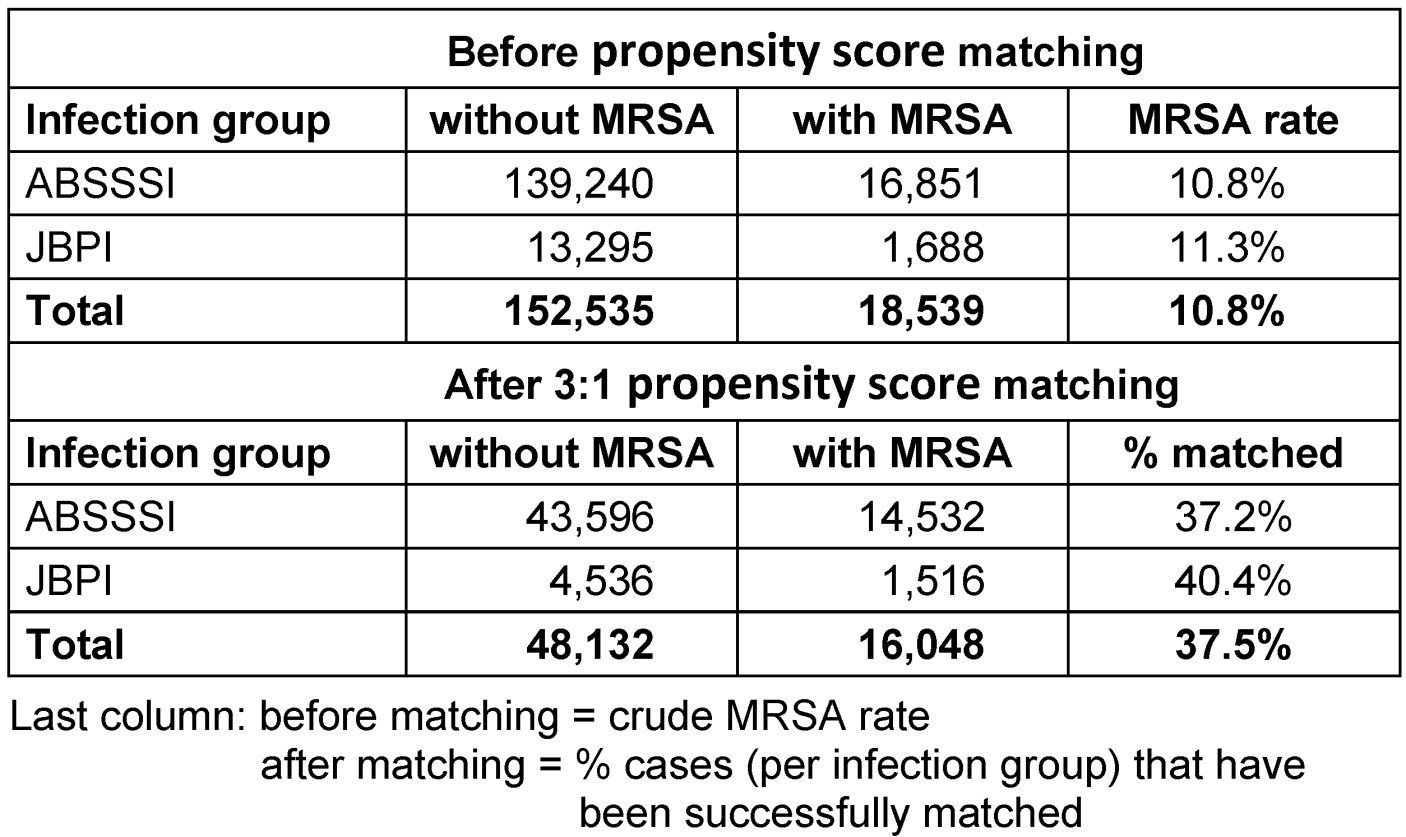
Disease entities, number of cases with and without MRSA infections before and after matching

**Table 3:**
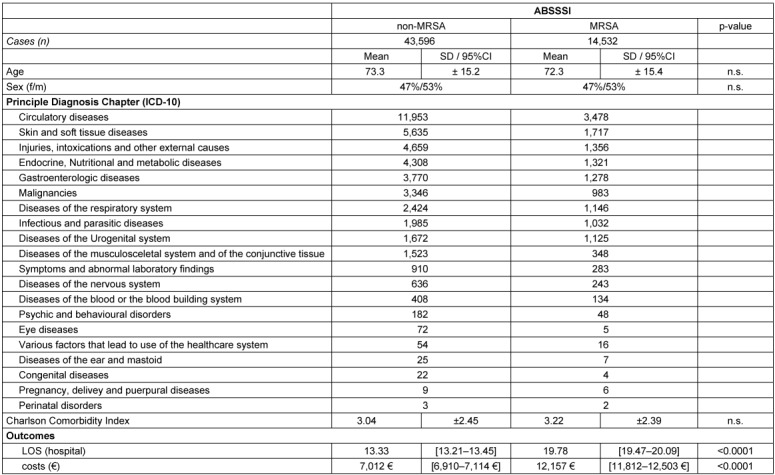
Descriptive statistics and outcomes in ABSSSI patients (matched sample)

**Table 4:**
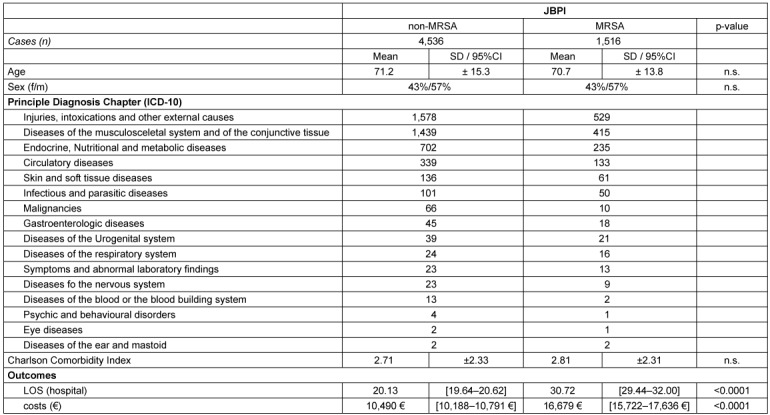
Descriptive statistics and outcomes in JBPI patients (matched sample)

**Table 5:**
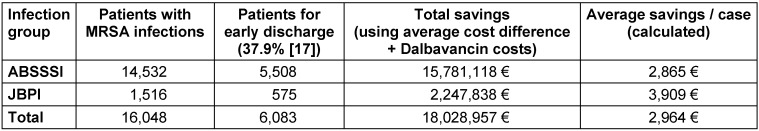
Summary of the savings calculation using the proportion of dischargeable patients as well as average cost difference between MRSA and non-MRSA infections adding Dalbavancin treatment costs

## References

[1] Rao N, Ziran BH, Lipsky BA (2011). Treating osteomyelitis: antibiotics and surgery. Plast Reconstr Surg.

[2] Lazzarini L, Lipsky BA, Mader JT (2005). Antibiotic treatment of osteomyelitis: what have we learned from 30 years of clinical trials?. Int J Infect Dis.

[3] Lener S, Hartmann S, Barbagallo GMV, Certo F, Thomé C, Tschugg A (2018). Management of spinal infection: a review of the literature. Acta Neurochir (Wien).

[4] Jenkins TC, Sabel AL, Sarcone EE, Price CS, Mehler PS, Burman WJ (2010). Skin and soft-tissue infections requiring hospitalization at an academic medical center: opportunities for antimicrobial stewardship. Clin Infect Dis.

[5] Nathwani D, Dryden M, Garau J (2016). Early clinical assessment of response to treatment of skin and soft-tissue infections: how can it help clinicians? Perspectives from Europe. Int J Antimicrob Agents.

[6] Walsh TL, Chan L, Konopka CI, Burkitt MJ, Moffa MA, Bremmer DN, Murillo MA, Watson C, Chan-Tompkins NH (2016). Appropriateness of antibiotic management of uncomplicated skin and soft tissue infections in hospitalized adult patients. BMC Infect Dis.

[7] Al-Omari A, Cameron DW, Lee C, Corrales-Medina VF (2014). Oral antibiotic therapy for the treatment of infective endocarditis: a systematic review. BMC Infect Dis.

[8] Martí-Carvajal AJ, Dayer M, Conterno LO, Gonzalez Garay AG, Martí-Amarista CE, Simancas-Racines D (2016). A comparison of different antibiotic regimens for the treatment of infective endocarditis. Cochrane Database Syst Rev.

[9] Anderson DJ, Kaye KS, Chen LF, Schmader KE, Choi Y, Sloane R, Sexton DJ (2009). Clinical and financial outcomes due to methicillin resistant Staphylococcus aureus surgical site infection: a multi-center matched outcomes study. PLoS ONE.

[10] Macedo-Viñas M, De Angelis G, Rohner P, Safran E, Stewardson A, Fankhauser C, Schrenzel J, Pittet D, Harbarth S (2013). Burden of meticillin-resistant Staphylococcus aureus infections at a Swiss University hospital: excess length of stay and costs. J Hosp Infect.

[11] Debatin JF, Ekkernkamp A, Schulte B, Baehr M (2010). Krankenhausmanagement: Strategien, Konzepte, Methoden.

[12] Jerosch J, Linke C (2016). Patientenzentrierte Medizin in Orthopädie und Unfallchirurgie: Lösungen für Patientenorientierung, Qualität und Wirtschaftlichkeit.

[13] Linke C, Jerosch J, Linke C (2016). Verweildauer als Zielparameter der Patientenversorgung aus ökonomischer und medizinischer Sicht. Patientenzentrierte Medizin in Orthopädie und Unfallchirurgie.

[14] Roeder N, Hensen P, Franz D (2014). Gesundheitsökonomie, Gesundheitssystem und öffentliche Gesundheitspflege: Ein praxisorientiertes Kurzlehrbuch.

[15] Bartkowski R (2012). Spital-Verweildauer unter DRG-Bedingungen. Ther Umsch.

[16] Cots F, Chiarello P, Salvador X, Quentin W, Busse R, Geissler A, Quentin W, Wiley M (2011). DRG-based hospital payment: intended and unintended consequences. Diagnosis related groups in Europe: moving towards transparency, efficiency and quality in hospitals.

[17] Eckmann C, Lawson W, Nathwani D, Solem CT, Stephens JM, Macahilig C, Simoneau D, Hajek P, Charbonneau C, Chambers R, Li JZ, Haider S (2014). Antibiotic treatment patterns across Europe in patients with complicated skin and soft-tissue infections due to meticillin-resistant Staphylococcus aureus: a plea for implementation of early switch and early discharge criteria. Int J Antimicrob Agents.

[18] Sharpe JN, Shively EH, Polk HC (2005). Clinical and economic outcomes of oral linezolid versus intravenous vancomycin in the treatment of MRSA-complicated, lower-extremity skin and soft-tissue infections caused by methicillin-resistant Staphylococcus aureus. Am J Surg.

[19] Itani KM, Dryden MS, Bhattacharyya H, Kunkel MJ, Baruch AM, Weigelt JA (2010). Efficacy and safety of linezolid versus vancomycin for the treatment of complicated skin and soft-tissue infections proven to be caused by methicillin-resistant Staphylococcus aureus. Am J Surg.

[20] Li JZ, Willke RJ, Rittenhouse BE, Rybak MJ (2003). Effect of linezolid versus vancomycin on length of hospital stay in patients with complicated skin and soft tissue infections caused by known or suspected methicillin-resistant staphylococci: results from a randomized clinical trial. Surg Infect (Larchmt).

[21] Arena F, Romanini E, Rosi E, Salomone C, Tucci G, Pempinello C, Fantoni M (2018). The role of dalbavancin in the multi-disciplinary management of wound infections in orthopaedic surgery. J Chemother.

[22] GKV-Spitzenverband, Verband der Privaten Krankenversicherung, Deutsche Krankenhausgesellschaft (2018). Vereinbarung zum Fallpauschalensystem für Krankenhäuser für das Jahr 2018 (Fallpauschalenvereinbarung 2018 – FPV 2018).

[23] InEK GmbH Fallpauschalenkatalog 2018.

[24] Felder S (2009). The variance of length of stay and the optimal DRG outlier payments. Int J Health Care Finance Econ.

[25] Köck R, Becker K, Cookson B, van Gemert-Pijnen JE, Harbarth S, Kluytmans J, Mielke M, Peters G, Skov RL, Struelens MJ, Tacconelli E, Navarro Torné A, Witte W, Friedrich AW (2010). Methicillin-resistant Staphylococcus aureus (MRSA): burden of disease and control challenges in Europe. Euro Surveill.

[26] Resch A, Wilke M, Fink C (2009). The cost of resistance: incremental cost of methicillin-resistant Staphylococcus aureus (MRSA) in German hospitals. Eur J Health Econ.

[27] Hübner C, Hübner NO, Hopert K, Maletzki S, Flessa S (2014). Analysis of MRSA-attributed costs of hospitalized patients in Germany. Eur J Clin Microbiol Infect Dis.

[28] Dunne MW, Puttagunta S, Sprenger CR, Rubino C, Van Wart S, Baldassarre J (2015). Extended-duration dosing and distribution of dalbavancin into bone and articular tissue. Antimicrob Agents Chemother.

[29] Office of Communications / Division of Drug Information (2013). Guidance for Industry: Acute Bacterial Skin and Skin Structure Infections: Developing Drugs for Treatment.

[30] Inmed Über uns.

[31] Bouza E, Valerio M, Soriano A, Morata L, Carus EG, Rodríguez-González C, Hidalgo-Tenorio MC, Plata A, Muñoz P, Vena A, DALBUSE Study Group (Dalbavancina: Estudio de su uso clinico en España) (2018). Dalbavancin in the treatment of different gram-positive infections: a real-life experience. Int J Antimicrob Agents.

[32] Juul JJ, Mullins CF, Peppard WJ, Huang AM (2016). New developments in the treatment of acute bacterial skin and skin structure infections: considerations for the effective use of dalbavancin. Ther Clin Risk Manag.

[33] Nair T, Fitzgerald J, Ly B, Wallace MR (2018). Dalbavancin as a cost effective antibiotic. Infect Dis (Lond).

[34] Statistisches Bundesamt (Destatis) (2017). Gesundheit: Fallpauschalenbezogene Krankenhausstatistik (DRG-Statistik). Diagnosen, Prozeduren, Fallpauschalen und Case Mix der vollstationären Patientinnen und Patienten in Krankenhäusern.

[35] Robert Koch-Institut (RKI) (2018). Eigenschaften, Häufigkeit und Verbreitung von MRSA in Deutschland. Krankenhhyg Infektionsverhütung.

[36] European Centre for Disease Prevention and Control (2017). Surveillance of antimicrobial resistance in Europe 2016: Annual report of the European Antimicrobial Resistance Surveillance Network (EARS-Net).

[37] Righi E, Carnelutti A, Vena A, Bassetti M (2018). Emerging treatment options for acute bacterial skin and skin structure infections: focus on intravenous delafloxacin. Infect Drug Resist.

[38] Falconer S, Laing, Robert Reduced hospital stays for skin infections treated with dalbavancin.

[39] Laupland KB, Valiquette L (2013). Outpatient parenteral antimicrobial therapy. Can J Infect Dis Med Microbiol.

[40] Tice AD, Rehm SJ, Dalovisio JR, Bradley JS, Martinelli LP, Graham DR, Gainer RB, Kunkel MJ, Yancey RW, Williams DN, IDSA (2004). Practice guidelines for outpatient parenteral antimicrobial therapy. IDSA guidelines. Clin Infect Dis.

[41] Kardas P (2002). Patient compliance with antibiotic treatment for respiratory tract infections. J Antimicrob Chemother.

[42] Falagas ME, Karagiannis AK, Nakouti T, Tansarli GS (2015). Compliance with once-daily versus twice or thrice-daily administration of antibiotic regimens: a meta-analysis of randomized controlled trials. PLoS ONE.

[43] Eells SJ, Nguyen M, Jung J, Macias-Gil R, May L, Miller LG (2016). Relationship between Adherence to Oral Antibiotics and Postdischarge Clinical Outcomes among Patients Hospitalized with Staphylococcus aureus Skin Infections. Antimicrob Agents Chemother.

[44] Nguyen-Van-Tam SE, Nguyen-Van-Tam JS, Myint S, Pearson JC (1999). Risk factors for hospital-acquired urinary tract infection in a large English teaching hospital: a case-control study. Infection.

[45] El-Masri MM, Hammad TA, McLeskey SW, Joshi M, Korniewicz DM (2004). Predictors of nosocomial bloodstream infections among critically ill adult trauma patients. Infect Control Hosp Epidemiol.

[46] Hernández A, Yagüe G, García Vázquez E, Simón M, Moreno Parrado L, Canteras M, Gómez J (2018). Infecciones nosocomiales por Pseudomonas aeruginosa multiresistente incluido carbapenémicos: factores predictivos y pronósticos. Estudio prospectivo 2016-2017. Rev Esp Quimioter.

[47] Allergan Pharmaceuticals International Ltd. (2015). Xydalba (Dalbavancin) EU SmPC_EN_Dec 2018: Summary of Product Characteristics. EU/1/14/986/001.

[48] Dunne MW, Talbot GH, Boucher HW, Wilcox M, Puttagunta S (2016). Safety of Dalbavancin in the Treatment of Skin and Skin Structure Infections: A Pooled Analysis of Randomized, Comparative Studies. Drug Saf.

[49] Tobudic S, Forstner C, Burgmann H, Lagler H, Ramharter M, Steininger C, Vossen MG, Winkler S, Thalhammer F (2018). Dalbavancin as Primary and Sequential Treatment for Gram-Positive Infective Endocarditis: 2-Year Experience at the General Hospital of Vienna. Clin Infect Dis.

[50] Chapman AL, Seaton RA, Cooper MA, Hedderwick S, Goodall V, Reed C, Sanderson F, Nathwani D, BSAC/BIA OPAT Project Good Practice Recommendations Working Group (2012). Good practice recommendations for outpatient parenteral antimicrobial therapy (OPAT) in adults in the UK: a consensus statement. J Antimicrob Chemother.

[51] Bowling JE, Lewis JS, Owens AD (2013). Outpatient Parenteral Antimicrobial Therapy. Hosp Med Clin.

[52] Buehrle DJ, Shields RK, Shah N, Shoff C, Sheridan K (2017). Risk Factors Associated With Outpatient Parenteral Antibiotic Therapy Program Failure Among Intravenous Drug Users. Open Forum Infect Dis.

[53] Paladino JA, Poretz D (2010). Outpatient parenteral antimicrobial therapy today. Clin Infect Dis.

