# Supplementary material for: Potential savings through single-dose intravenous Dalbavancin in long-term MRSA infection treatment – a health economic analysis using German DRG data
Source: GMS Infect Dis. 2019 Oct 23;7:Doc03. doi: 10.3205/id000043 (PMC6839362; doi:10.3205/id000043)
Supplement: Definitions for infection groups and infections with corresponding ICD-10 codes [file ID-07-03-s-001.pdf]

Attachment 1 to: Wilke M, Worf K, Preisendörfer B, Heinlein W, Kast T, Bodmann KF. Potential savings through single-dose intravenous Dalbavancin in long-term MRSA infection treatment – a health economic analysis using German DRG data. GMS Infect Dis. 2019;7:Doc03. DOI: 10.3205/id000043

Definitions for infection groups and infections with corresponding ICD-10 codes

**Infection Group / Infection / ICD-10-Code + Text**
**ABSSSI**
**Cellulitis/erysipelas**
L00.0: Staphylococcal scalded skin syndrome [SSS syndrome]: infestation of less than 30% of the body surface
L00.1: Staphylococcal scalded skin syndrome [SSS syndrome]: infestation of 30% of the body surface and more
L00: Staphylococcal scalded skin syndrome
L03.01: Cellulitis of finger
L03.02: Cellulitis of toe
L03.10: Cellulitis of upper limb
L03.11: Cellulitis of lower limb
L03.2: Cellulitis of face
L03.3: Cellulitis of trunk
L03.9: Cellulitis, unspecified
L03: Cellulitis
L04.3: Acute lymphadenitis of lower limb
L08.8: Other specified local infections of skin and subcutaneous tissue
L08.9: Local infection of skin and subcutaneous tissue, unspecified
**Major cutaneous abscess**
K61.0: Anal abscess
K61.1: Rectal abscess
K61.2: Anorectal abscess
K61.3: Ischiorectal abscess
K61.4: Intrasphincteric abscess
K61: Abscess in the anal and rectal region
L02.0: Cutaneous abscess, furuncle and carbuncle of face
L02.1: Cutaneous abscess, furuncle and carbuncle of neck
L02.2: Cutaneous abscess, furuncle and carbuncle of trunk
L02.3: Cutaneous abscess, furuncle and carbuncle of buttock
L02.4: Cutaneous abscess, furuncle and carbuncle of limb
L02.8: Cutaneous abscess, furuncle and carbuncle of other sites
L02.9: Cutaneous abscess, furuncle and carbuncle, unspecified
L02: Skin abscess, furun and carbuncle
L05.0: Pilonidal cyst with abscess
**Wound infection**
E10.74: Diabetes mellitus, type 1: with multiple complications: With diabetic foot syndrome, not referred to as derailed
E10.75: Diabetes mellitus, type 1: With multiple complications: With diabetic foot syndrome, designated as derailed
E11.74: Diabetes mellitus, type 2: with multiple complications: with diabetic foot syndrome, not referred to as derailed
E11.75: Diabetes mellitus, type 2: with multiple complications: With diabetic foot syndrome, designated as derailed
I70.24: Atherosclerosis of arteries of extremities with gangrene
I70.25: Atherosclerosis of the extremity arteries: pelvic-leg type, with gangrene
I83.0: Varicose veins of lower extremities with ulcer
I83.2: Varicose veins of lower extremities with both ulcer and inflammation
L89.1: Decubitus of 2nd degree
L89.2: Decubitus of the 3rd degree
L89.3: Decubitus of the 4th degree

L89.9: Decubitus, degree unspecified
L97: Ulcer of lower limb, not elsewhere classified
I98.4: Chronic ulcer of skin, not elsewhere classified
R02: Gangrene, not elsewhere classified
T79.3: Post-traumatic wound infection, not elsewhere classified
T81.4: Infection following a procedure, not elsewhere classified
T87.4: Infection of amputation stump
**Joint and Prostheses Infections**
**Infection of joint prostheses**
T84.5: Infection and inflammatory reaction due to internal joint prosthesis
**Infection of osteosyntheses materials**
T84.6: Infection and inflammatory reaction due to internal fixation device [any site]
**Infection other orthopedic prosthesis**
T84.7: Infection and inflammatory reaction due to other internal orthopaedic prosthetic devices, implants and grafts
**Joint abscess**
M00.81: Arthritis and polyarthritis due to other specified bacterial agents, shoulder region
M00.82: Arthritis and polyarthritis due to other specified bacterial agents, upper arm
M00.83: Arthritis and polyarthritis due to other specified bacterial agents, forearm
M00.84: Arthritis and polyarthritis due to other specified bacterial agents, hand
M00.85: Arthritis and polyarthritis due to other specified bacterial agents, pelvic region and thigh
M00.86: Arthritis and polyarthritis due to other specified bacterial agents, lower leg
M00.87: Arthritis and polyarthritis due to other specified bacterial agents, ankle and foot
M00.88: Arthritis and polyarthritis due to other specified bacterial agents, other site
M00.89: Arthritis and polyarthritis due to other specified bacterial agents, site unspecified
M00.90: Pyogenic arthritis, unspecified, multiple sites
M00.91: Pyogenic arthritis, unspecified, shoulder region
M00.92: Pyogenic arthritis, unspecified, upper arm
M00.93: Pyogenic arthritis, unspecified, forearm
M00.94: Pyogenic arthritis, unspecified, hand
M00.95: Pyogenic arthritis, unspecified, pelvic region and thigh
M00.96: Pyogenic arthritis, unspecified, lower leg
M00.97: Pyogenic arthritis, unspecified, ankle and foot
M00.98: Pyogenic arthritis, unspecified, other site
M00.99: Pyogenic arthritis, unspecified, site unspecified
M00: Purulent arthritis
**Osteomyelitis**
M86: Osteomyelitis
